# Imaging the *ex-vivo* human cochlea using 1.3-*μ*m and 1.7-*μ*m optical coherence tomography

**DOI:** 10.1117/1.JBO.30.4.046007

**Published:** 2025-04-17

**Authors:** Jack C. Tang, Dorothy W. Pan, John S. Oghalai, Brian E. Applegate

**Affiliations:** a University of Southern California, Caruso Department of Otolaryngology—Head and Neck Surgery, Los Angeles, California, United States; b University of Southern California, Alfred Mann Department of Biomedical Engineering, Los Angeles, California, United States; c University of Southern California, Ming Hsieh Department of Electrical and Computer Engineering, Los Angeles, California, United States

**Keywords:** optical coherence tomography, otolaryngology, otology, inner ear, cochleae

## Abstract

**Significance:**

There is no clinical imaging method to visualize the soft tissues of the human cochlea, which are crucial for sound transduction and are damaged in sensorineural hearing loss. Although optical coherence tomography (OCT) has been effective in small animal models, we show for the first time that it can image through the full thickness of the *ex-vivo* human otic capsule and resolve cochlear microstructures despite increased scattering.

**Aim:**

We aim to investigate whether OCT could image the cochlea through the otic capsule. We compared 1.7 and 1.3  μm OCT to test if the reduced scattering at 1.7  μm provided any appreciable advantage for imaging the cochleae.

**Approach:**

OCT interferometers were built for both 1.3 and 1.7  μm wavelengths, using identical sample and reference arm optics in both systems. Imaging was performed on two fixed human temporal bones with intact cochleae. The interferometers were designed to allow seamless switching between 1.3 and 1.7  μm OCT without disrupting the temporal bone during imaging.

**Results:**

We took volumetric OCT images at the base, apex, and hook regions of fixed *ex-vivo* human cochleae and compared the images taken at 1.3  μm with those taken at 1.7  μm. At both wavelengths, we could see through the otic capsule and identify cochlear structures. In some cases, 1.7  μm OCT resulted in clearer images of the lateral wall, interior scala, and fine cochlear structures due to reduced multiple scattering at depth compared with 1.3  μm.

**Conclusions:**

We conclude that both 1.7  μm and 1.3  μm OCT can image through the human otic capsule, offering the potential for direct measurement of cochlear vibrometry or blood flow in living humans. Using 1.7  μm light, we observed reduced multiple scattering in the otic capsule, leading to enhanced contrast of cochlear structures compared with 1.3  μm. However, these improvements were marginal and came with trade-offs.

## 1 Introduction

Hearing loss impacts the quality of life of nearly a quarter of adults in the United States.^1^ Many types of hearing loss directly affect the cochlea, including noise-induced hearing loss, presbycusis (age-related hearing loss), and congenital hearing loss. The unique cochlear anatomy makes it challenging to image the small structures deep within the dense bone of the otic capsule. Currently, there is an unmet need for medical imaging modalities that are capable of both the high resolution and imaging depth necessary to image the intact human cochlea.

Optical coherence tomography (OCT) and its functional extensions can potentially be used to measure the morphology, vibratory function, and blood flow perturbations in the cochlea^2^^,^^3^ and are becoming increasingly relevant for hearing research. In recent years, many groups have used OCT for hearing research in mice and other small animal models. Mice have been particularly valuable, primarily due to their suitability for genetic modeling of specific hearing deficits, allowing researchers to investigate the precise mechanisms of dysfunction. These efforts are generating a wealth of information on the structure and function of the inner ear, including high-resolution morphology,^4^ vibrometry,^3^^,^^5^^–^^10^ and angiography.^2^^,^^3^

In humans, the otic capsule is significantly thicker than in rodents, measuring 500 to 1000  μm compared with 50 to 200  μm in mice. This increased thickness presents a greater challenge for *in-vivo* imaging. Different OCT systems have been built specifically for imaging the human ear, including surgical microscopes,^5^ handheld otoscopes,^11^^,^^12^ catheter-based,^13^^,^^14^ and endoscopic systems.^15^ There is a growing body of promising work that uses OCT to probe otopathologies in the human middle ear, but *in-vivo* imaging of the cochlea is still elusive; the increased otic capsule thickness in humans has proven challenging to overcome. The possibility of OCT imaging through the full thickness of the otic capsule has remained uncertain until now. Short-wave IR light at 1.3  μm has increased imaging depth for OCT (compared with shorter wavelength visible and near-IR light) due to lower scattering in tissue compared with near-infrared light. This comes with a slight penalty due to water absorption. Using 1.3  μm OCT, our group was able to non-invasively image through the eardrum and see into the living cochlea, resolving what appeared to be the edge of the lateral wall (Fig. 9 in Ref. 5). However, we were unable to see any identifiable soft tissue structures within the cochlea and the lateral wall appeared blurry due to multiple scattering of photons through the thickness of bone. Therefore, to provide the additional imaging depth needed to access the human cochlear soft tissues, we sought to use 1.7  μm due to its lower scattering coefficient in bone compared with 1.3  μm. ^16^ Based on Eq. 1 of Ref. 16 and the mean values in Table 2, we expect the reduced scattering coefficient $\mu_{s}^{\prime }$ in the bone to be ∼17.6% lower at 1.7  μm ($9.53\;{\mathrm{cm}}^{-1}$) than at 1.3  μm ($11.56\;{\mathrm{cm}}^{-1}$). However, there is a large variation in the measured scattering coefficient for the bone,^16^^–^^19^ such that if we compare each set of parameters for bone in reference,^16^ we find a range of 3.7 to 32.6%. The four curves are shown in Fig. S1 in the Supplementary Material for reference. Nevertheless, long-wavelength OCT at 1.7  μm has been used to provide additional imaging depth in a variety of different tissue types,^20^^–^^23^ and we expected similar performance in the otic capsule due to the lower scattering coefficient in bone as wavelength increases.^16^ Thus, we constructed a dual-wavelength OCT system using 1.3  μm and 1.7  μm to demonstrate the differences for imaging the *ex-vivo* human cochlea.

## 2 Materials and Methods

### 2.1 OCT System Configuration for 1.3 *μ*m and 1.7 *μ*m

**Fig. 1:**
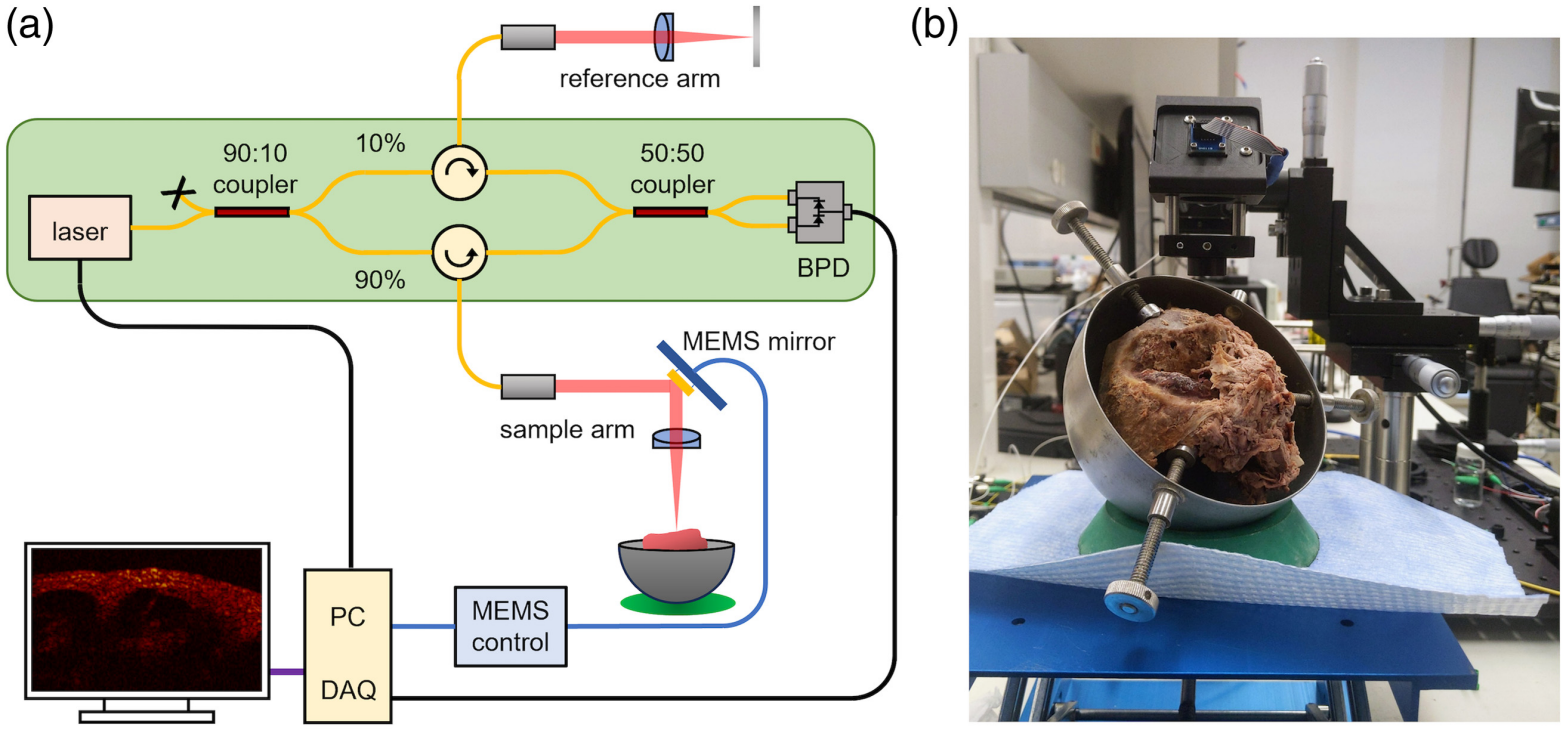
Instrumentation for OCT imaging of *ex-vivo* human temporal bones. (a) Schematic diagram of the Mach–Zehnder OCT interferometers that were used for imaging. (b) Human temporal bone positioned under the OCT sample arm. We coarsely positioned the temporal bones using a vertical jack stand (blue) and finely adjusted the position of the OCT sample arm using a three-axis translation stage (black). Abbreviations: BPD, balanced photodetector; PC, personal computer; DAQ, data acquisition board; MEMS, microelectromechanical system.

We constructed an OCT system that could easily be switched between 1.3 and 1.7  μm lasers without disturbing the sample. To do this, we constructed two Mach–Zehnder OCT interferometers (one for each laser) that could be swapped to use the same sample arm and reference arm paths. The first OCT interferometer used a 1.3-μm akinetic swept-source laser from Insight Photonic Solutions (SLE-101) and the second OCT interferometer used a 1.7-μm swept-source laser from Santec Holdings Corp. (HSL-40-90B). We show a schematic diagram of the OCT system in Fig. 1(a). Each OCT interferometer used fiber components and balanced photodetectors that were specific to the wavelength used. This allowed us to quickly switch the OCT system from 1.3 to 1.7  μm (and vice versa) by interchanging the laser interferometers without moving the sample.

In the schematic diagram of the combined 1.3- and 1.7-μm OCT system shown in Fig. 1(a), the light-green region indicates the fiber-based Mach–Zehnder interferometer that is replaced when changing between 1.3- and 1.7-μm OCT imaging configurations. In both the sample and reference arms, we used a fixed-focus collimator (F240APC-1550, Thorlabs, Inc., Newton, New Jersey, United States) and a ½″ achromatic doublet (f=50  mm, AC127-050-C, Thorlabs, Inc.) as a focusing lens. Using the same optical elements allowed us to minimize any difference in dispersion between the sample and reference arms. In addition to these optics, the sample arm also had a MEMS scanning mirror (Mirrorcle Technologies, Inc., Richmond, Virginia, United States) between the collimator and the ½″ achromatic doublet that allowed us to scan a $10\times 10\;{\mathrm{mm}}^{2}$ field of view. There were subtle differences in the lateral and axial resolution due to the different wavelengths and bandwidths of the 1.3 and 1.7  μm lasers. The 1.7-μm OCT had a slightly worse lateral resolution than the 1.3-μm OCT, 66.2 versus 56.5  μm, respectively, but a slightly better axial resolution, 11.7 versus 12.9  μm (n=1.4), respectively, due to its wider bandwidth. The coherence length of the 1.7-μm laser was also much shorter than the 1.3-μm laser (15.1 versus >1000  mm). Figure 1(b) shows the orientation of the temporal bone under the OCT sample arm. The temporal bone was coarsely positioned on the blue vertical jack stand, whereas precise adjustments to the OCT sample arm were made using a manual XYZ translation stage.

### 2.2 Temporal Bone Imaging

We obtained two formaldehyde-fixed frozen cadaveric human temporal bones from the USC Anatomical Gift Program and thawed them at 4°C prior to dissection and OCT imaging. These temporal bones were previously used for general anatomy dissections, but the cochleae remained intact. We expect that formaldehyde-based fixation will not change the tissue refractive index appreciably,^24^^,^^25^ but will increase the scattering coefficient due to the formation of inter-molecular cross-links between macromolecules.^26^^–^^28^ An increased scattering coefficient should nominally reduce overall imaging depth but could also improve contrast within the image compared with fresh tissue. We removed extraneous tissue from the temporal bones and fenestrated one of the cochleae so that the locations of the apical and basal turns could be easily seen; we refer to this specimen as temporal bone 1 (TB1). Another cochlear specimen was left fully intact (TB2). We then used the above system to acquire volumetric images using the 1.3-μm OCT, then switched to using the 1.7-μm OCT without disturbing the position of the specimen.

Throughout this paper, we show 2D images generated by rotating and slicing the OCT volumes with Amira Software (Thermo Fisher Scientific). This is similar to other medical imaging workflows such as CT and MRI, in which 3D imaging allows the alignment of the imager/subject to be relatively tolerant of error, and the volumetric data are used to more precisely reconstruct 2D images of the structures of interest. Volumetric imaging will be essential for OCT otoscopy because it allows users to rapidly scan and capture OCT data from the entire field of view. This makes for easier alignment of an OCT otoscope inside the ear canal and can reduce procedure time and patient discomfort during imaging.

In Fig. S2 in the Supplementary Material, we show an illustration of the human cochlea with the approximate imaging angles for TB1 and TB2 (A and B, respectively). In TB1, we imaged the helicotrema along the central axis of the modiolus because we could easily locate it due to the fenestrations exposing parts of the apical and basal turns. We applied 1X phosphate-buffered saline (PBS) to TB1 to ensure that the fenestrated cochlea was filled with fluid during imaging. In the intact TB2, we imaged the base and hook regions of the cochlea from an approximate angle that would be visible during OCT otoscopy via the ear canal. The angle was approximated based on our experience imaging the middle ear through the ear canal with OCT.^5^^,^^11^^,^^12^ Key landmarks, such as the stapes (S), round window (RW) niche, and the cochlear promontory (P), may also be visible during OCT imaging via the ear canal.^5^^,^^12^ From the ear canal approach, the promontory is curved such that a view normal to the beam (minimized pathlength through the bone) is possible.^5^ Images from both TB1 and TB2 were of similar quality, and we were able to image structures inside the lateral wall of both cochleae.

### 2.3 Data Processing

The interferograms acquired from the OCT systems were processed using the standard method for swept-source OCT data (zero-padded fast Fourier transform, dispersion compensation, and Hann windowing). We used a refractive index of 1.4 for processing, which is a mid-range value for the refractive index of fixed temporal bone tissue.^15^ Because of differences in the laser power (∼30 versus ∼7  mW for 1.3 and 1.7  μm, respectively) and dynamic range of the balanced photodetectors, the 1.7-μm system had a lower signal-to-noise ratio (SNR) compared with the 1.3-μm system. We compared the performance of the 1.7- and 1.3-μm systems for both a single volume acquisition and an averaged volume acquisition where the image SNR was the same as that of 1.3  μm (n=12 volumes using 1.7  μm). For this comparison, we considered the SNR at the surface of the otic capsule.

We encountered some fixed pattern noise from the 1.7-μm system, ostensibly due to 1.7  μm being at the edge of the designated wavelength range for the sample and reference arm optics (Thorlabs C-coating). In comparison, 1.3  μm falls squarely in this range, and our 1.3  μm volumes did not suffer from this. Therefore, to remove the fixed pattern noise from the 1.7-μm volumes, we collected five background volumes at 1.7  μm (i.e., with no sample) and subtracted their average from the cochlea volumes acquired at 1.7  μm.

## 3 Results and Discussion

### 3.1 Apex Helicotrema

**Fig. 2:**
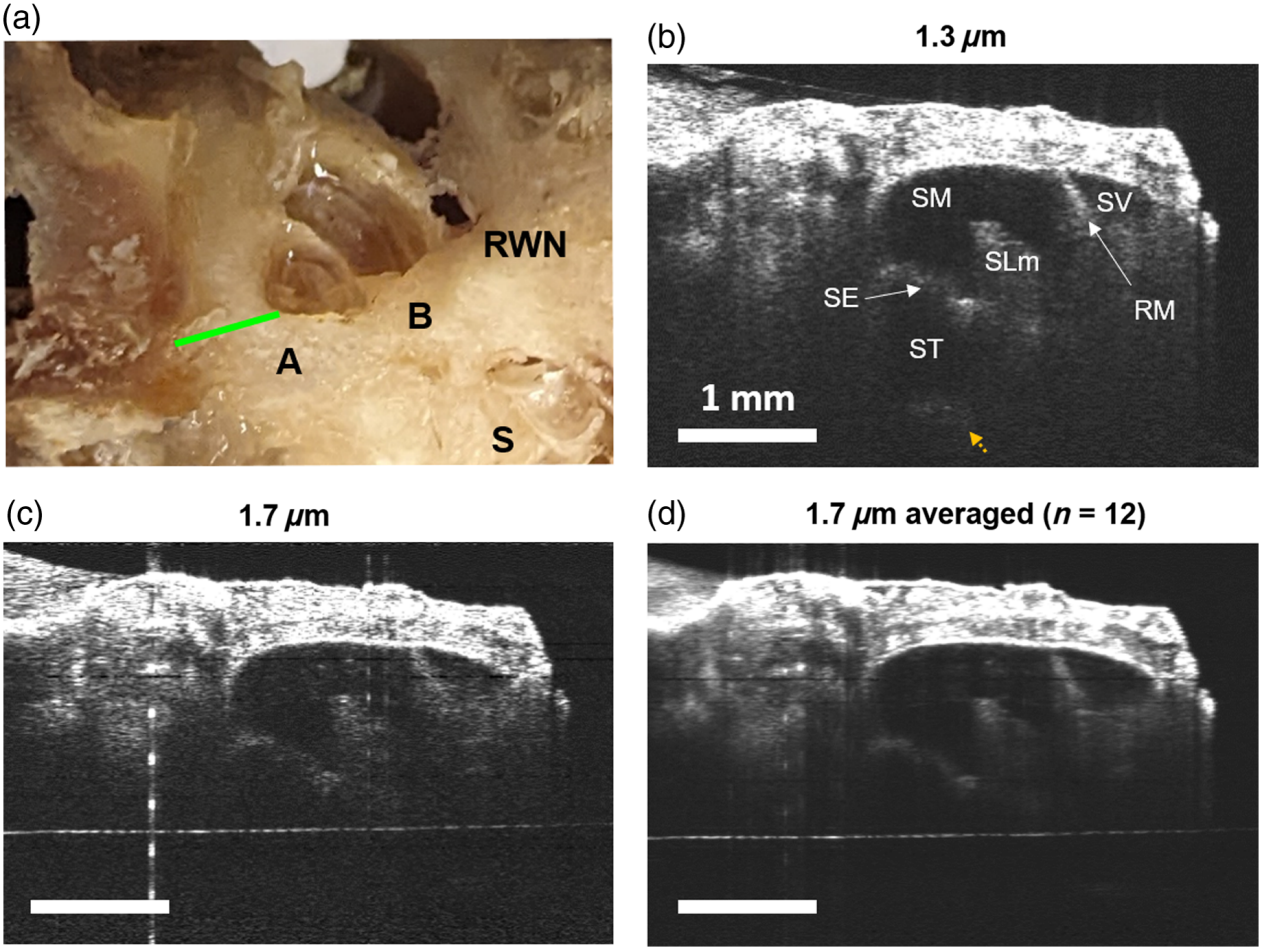
Imaging of the apical turn of a fixed human cochlea near the helicotrema. (a) TB1 shown with fenestrations at the apical (A) and basal (B) turns. The round window niche (RWN) and the stapes are also visible The location of the OCT cross-section is shown in green. (b) Cross-section of the apical turn taken using 1.3  μm OCT. Small anatomical features were visible in both 1.3 and 1.7  μm images, including Reissner’s membrane (RM), sensory epithelium (SE), spiral limbus (SLm), scala media (SM), scala tympani (ST), and scala vestibuli (SV). The separation between apical and basal turns could be seen at the bottom (yellow-dotted arrow). (c) OCT cross-section taken using 1.7  μm. (d) Averaged 1.7  μm cross-section (SNR normalized to that of 1.3  μm). Scale bars are 1 mm.

We first imaged TB1 at the apical turn, near the helicotrema where low-frequency sounds are transduced into neural signals in the cochlea. Because the cochlea was fenestrated, this helped us position the cochlea so that we could image the helicotrema along the central axis of the modiolus. This results in a similar cochlear view as we see in mice, with the apex at the top of the image which provides the ability to detect transverse vibrations in the organ of Corti.^6^^,^^29^^,^^30^ We applied 1X phosphate-buffered saline (PBS) to wet the surface of the cochlea and fill the helicotrema with fluid before imaging. In Fig. 2(a), we show a photograph of TB1 that shows the fenestrations drilled into the cochlea, which expose both the apical and basal turns (labeled A and B, respectively). We recorded volumetric images using 1.3 and 1.7  μm OCT and presented the cross-sections as Figs. 2(b) and 2(c). Using both 1.3 and 1.7  μm, we could image through the entire thickness of the otic capsule and identify structures inside the apical turn. The structures separating scala vestibuli (SV), scala media (SM), and scala tympani (ST) were all visible, including Reissner’s membrane (RM) and the sensory epithelium (SE). The spiral limbus (SLm) was also readily apparent using both wavelengths. In Fig. 2(c), we averaged 12 volumes from the 1.7-μm system to bring the overall image SNR up to that of the 1.3-μm image.

We found that using the 1.3-μm system, we could see the bone separating the apical and basal turns (yellow-dotted arrow). We were not able to see this at 1.7  μm, likely due to the much shorter coherence length of the 1.7-μm laser compared with the 1.3-μm laser (15.1 versus >1000  mm).

### 3.2 Basal Turn

**Fig. 3:**
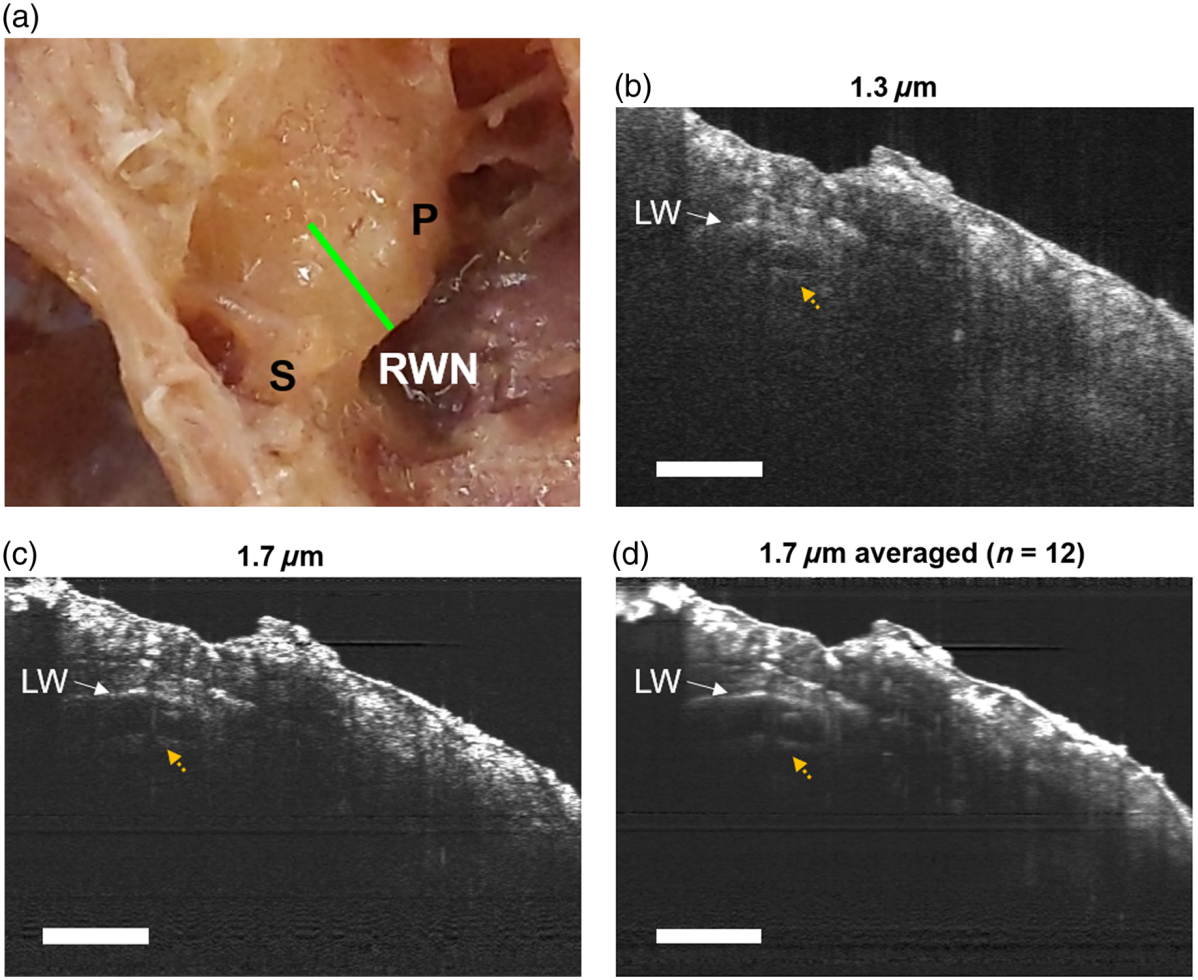
Imaging of the basal turn of a fixed human cochlea. (a) TB2 shown with the stapes (S), promontory (P), and round window niche (RWN) visible. The approximate location of the OCT cross-section is shown in green. (b) Cross-section of the basal turn using 1.3  μm OCT showing the lateral wall (LW) and part of the cochlear partition (yellow-dotted arrow). (c) OCT cross-section taken using 1.7  μm. (d) Averaged 1.7-μm cross-section (SNR normalized to that of 1.3  μm). Scale bars are 1 mm.

The basal region is where high-frequency sounds are transduced into neural signals. The base and the hook regions are located directly opposite the tympanic membrane when viewed through the ear canal, which may be accessible using otoscopic OCT *in vivo*. For this *ex-vivo* study, we chose a similar angle that approximates the otoscopic ear canal approach. In Fig. 3, we show a photograph and OCT cross-sections of TB2 at the basal turn from this angle. The location of the cross-section is indicated by the green line in Fig. 3(a). Using both 1.3 and 1.7  μm, we could clearly see the location of the lateral wall (LW), demonstrating that OCT can be used to image through the full thickness of the otic capsule at the cochlear base.

When comparing the 1.3-μm image [Fig. 3(b)] to both the single-acquisition and SNR-normalized 1.7  μm images [Figs. 3(c) and 3(d)], we observed reduced background noise within the otic capsule thickness due to decreased multiple scattering at 1.7  μm. This reduction in scattering resulted in higher contrast for the bony microstructure. There was also less background noise inside the scala at 1.7  μm and structures of the cochlear partition were clearer as a result (dotted yellow arrow), though we were unable to precisely identify these structures.

### 3.3 Hook Region

**Fig. 4:**
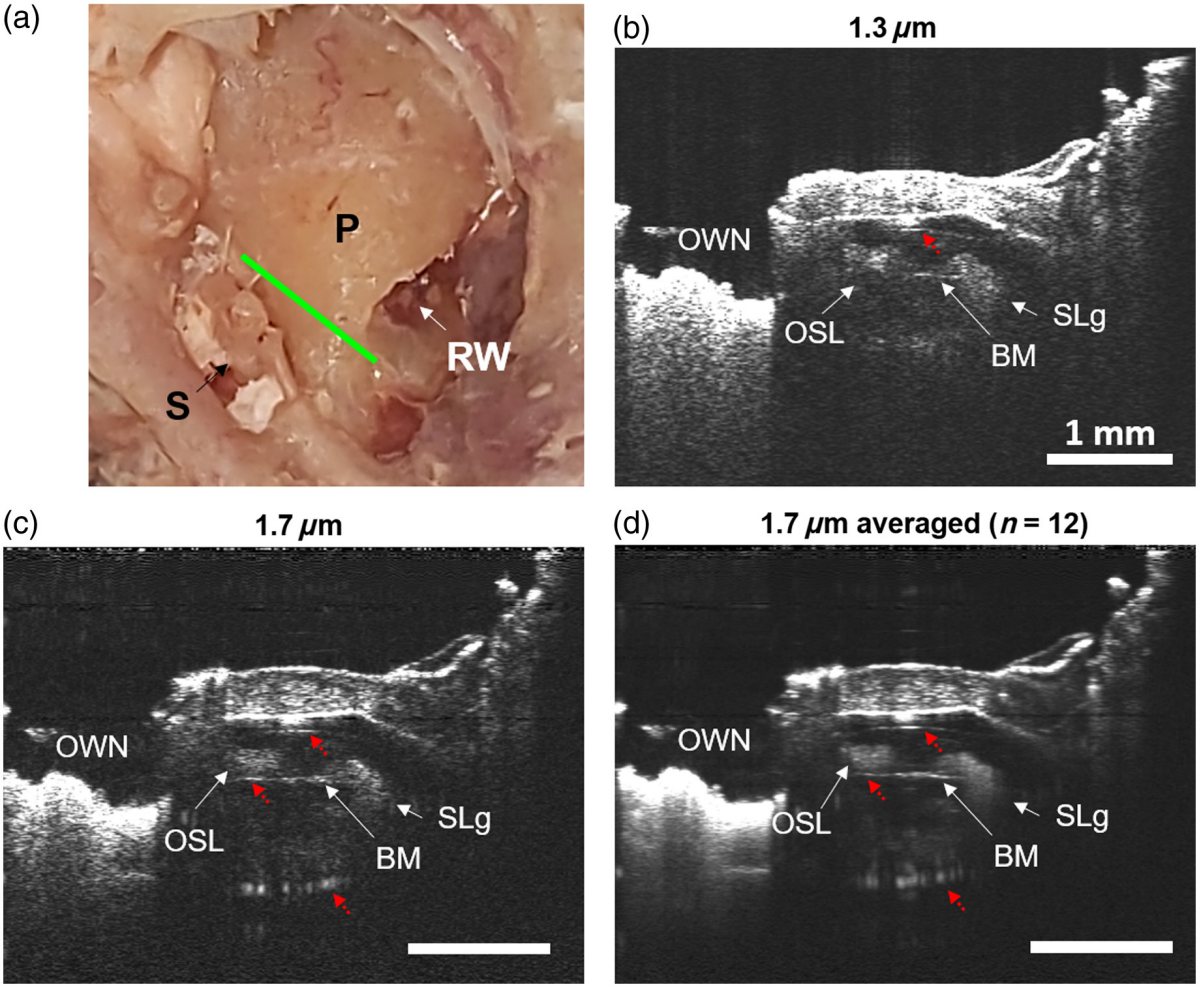
Imaging of the hook region of a fixed human cochlea. (a) View of TB2 shown with the stapes (S), promontory (P), and round window (RW) visible. The approximate location of the OCT cross-section is shown in green. (b) Cross-section of the hook region using 1.3  μm OCT. (c) OCT cross-section taken using 1.7  μm. (d) Averaged 1.7-μm cross-section (SNR normalized to that of 1.3  μm). At both 1.3 and 1.7  μm, the oval window niche (OWN), osseous spiral lamina (OSL), basilar membrane (BM), and spiral ligament (SLg) are visible. Scale bars are 1 mm.

The hook region is where the middle ear and the inner ear meet and has clinical relevance for cochlear implant surgery.^15^^,^^31^^–^^33^ Similar to the base, the hook is another potential area to be imaged via the ear canal with otoscopic OCT. In live human volunteers, we have been able to see this area, including the cochlear promontory and stapes through the intact tympanic membrane.^11^^,^^12^ In Fig. 4(a), we show a photograph and the location of our OCT cross-sections at the hook region in TB2. The resulting OCT volumes were similar in image quality to that of TB1. As with the base and the apex, we were also able to see through the full thickness of the otic capsule at this location as well, using both 1.3 and 1.7  μm [Figs. 4(b) and 4(c)]. We were also able to see inside the cochlea and identify the osseous spiral lamina (OSL), basilar membrane (BM), and spiral ligament (SLg). The oval window niche (OWN) was also in view at this angle, which is relevant for assessing patients undergoing stapedectomy.^34^^,^^35^ In this instance, we also saw imaging artifacts (labeled with red dotted arrows) that were repeated echoes from strong reflections at the bone–air interfaces at the promontory surface and lateral wall of the otic capsule. This artifact was more severe at 1.7 than at 1.3  μm, as shown by the additional imaging artifacts in Figs. 4(c) and 4(d).

Regarding potential differences in image quality between TB1 and TB2, both specimens were fixed using a formaldehyde-based fixative. In the case of TB1, the cochlea was filled with 1X PBS that was applied after opening the fenestrations in the base and apex. In TB2, the cochlea was intact and therefore remained filled with the fixative. The refractive index of the fixative inside the cochlea of TB2 is higher than 1X PBS, so the mismatch (and therefore contrast) should be slightly worse in TB2, but in our experiment, this did not result in an appreciable difference between the two specimens. As mentioned earlier, we anticipate that formaldehyde-based fixation will have minimal impact on the tissue refractive index^24^^,^^25^ but will increase the scattering coefficient due to inter-molecular cross-link formation between macromolecules.^26^^–^^28^

Generally, we positioned the temporal bone specimens so that the surface of the otic capsule was normal to the OCT beam. This both minimizes the pathlength of bone in the way of imaging and emulates the available *in-vivo* imaging approaches (ear canal or mastoidectomy). This general imaging method could tolerate modest adjustments in angle/position without losing the ability to image cochlear structures within, but gross changes in angle away from this normal condition introduced significantly more bone into the optical path and degraded our ability to image inside the cochlea. In the case of TB2, the angle at which we image the hook region and basal turn is close to what would be achievable *in vivo* via the ear canal, and the cochlear promontory is curved such that a view normal to the imaging beam is possible.^5^

## 4 Conclusion

We used 1.7 and 1.3  μm OCT to image fixed human temporal bones and found that both wavelengths were able to image through the full thickness of the otic capsule and resolve microanatomical structures inside the cochlea. Although the 1.7-μm laser was able to provide additional contrast and less multiple-scattering noise at depth, it did not increase the overall imaging depth and introduced reflection artifacts that were not present when using the 1.3-μm laser. Altogether, these data argue for the feasibility of using OCT for clinical diagnostic imaging of the human cochlea. For most clinical purposes, this would need to be done in the clinic on awake patients by imaging down their ear canal. The OCT system would need to peer through both the tympanic membrane and the full thickness of the human otic capsule. Further work is needed to determine whether this is indeed possible. Nevertheless, this work demonstrates that the otic capsule bone alone is not a prohibitive barrier to human cochlear imaging with OCT.

## Supplementary Material

10.1117/1.JBO.30.4.046007.s01

## Data Availability

OCT volume images used in this work are available on Mendeley Data (https://www.doi.org/10.17632/f48rxnrnw6.1).^36^ No specialized code was used for this research.
