# Supplementary material for: Imaging the ex-vivo human cochlea using 1.3-μm and 1.7-μm optical coherence tomography
Source: J Biomed Opt. 2025 Apr 17;30(4):046007. doi: 10.1117/1.JBO.30.4.046007 (PMC12005953; doi:10.1117/1.JBO.30.4.046007)
Supplement: Supplementary file 1 [file JBO_030_046007_SD001.docx]

# Supplementary Figures


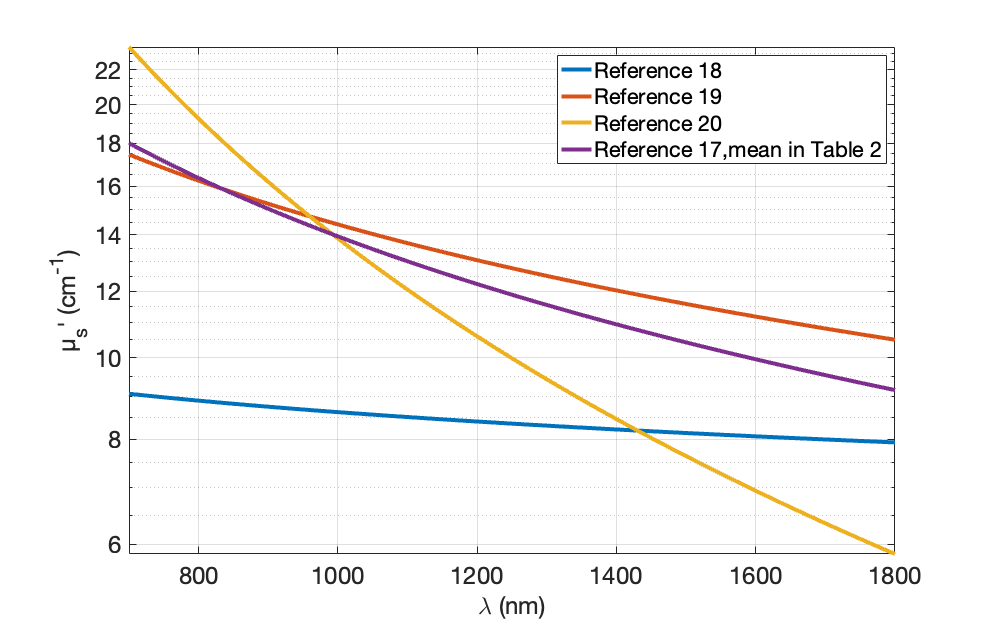


**Fig. S1**: Plots of the reduced scattering coefficient versus wavelength in the near IR to short-wave IR region. Plots are based on Equation 1 in Reference 17. Parameters for Equation 1 are from fits in reference 17 to data in references 18-20 and the mean values from the fits in reference 17.


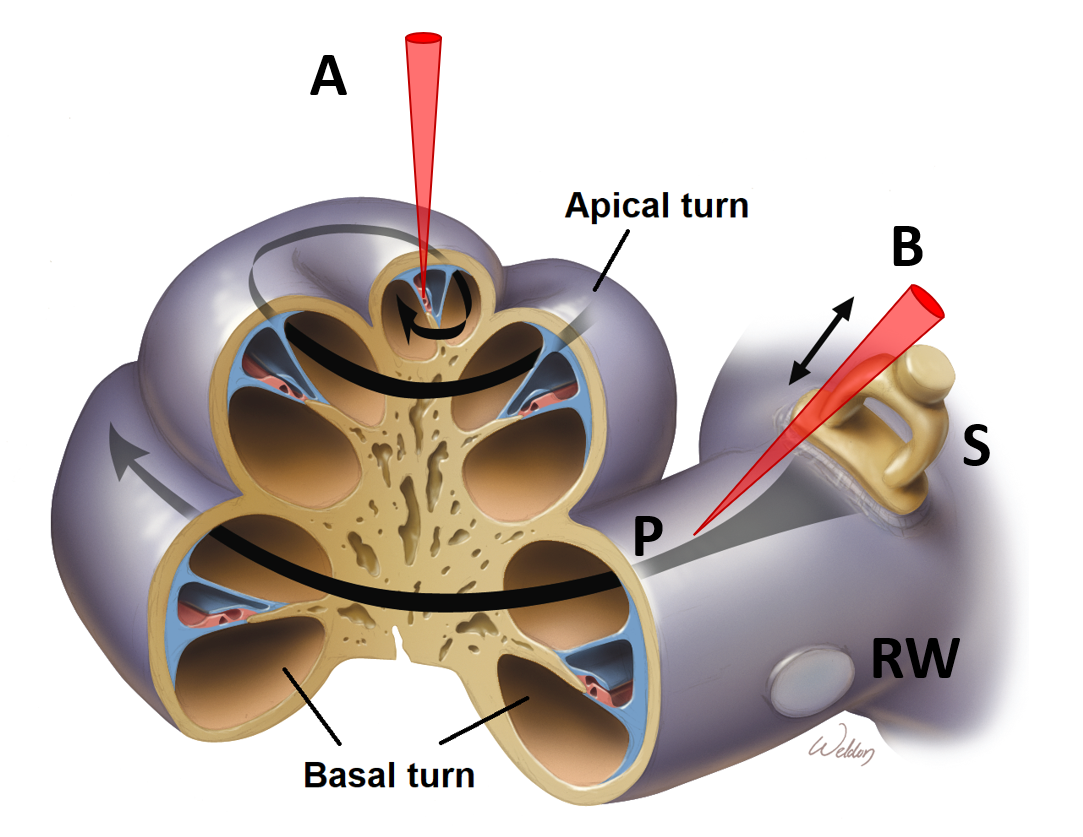


Fig. S2: Annotated illustration of the human cochlea. The approximate imaging angles (red cones) for TB1 and TB2 are labeled as A and B, respectively. The apical and basal turns of TB1 are fenestrated similar to the illustration. The stapes (S), round window (RW), and cochlear promontory (P) are key landmarks in the middle/inner ear region. Black arrows indicate the movement of the stapes and the direction of fluid wave propagation inside the cochlea due to sound.
